# Effects of Gelatin Methacrylate Bio-ink Concentration on Mechano-Physical Properties and Human Dermal Fibroblast Behavior

**DOI:** 10.3390/polym12091930

**Published:** 2020-08-26

**Authors:** Ming-You Shie, Jian-Jr Lee, Chia-Che Ho, Ssu-Yin Yen, Hooi Yee Ng, Yi-Wen Chen

**Affiliations:** 1 School of Dentistry, China Medical University, Taichung City 40447, Taiwan; eric@mail.cmu.edu.tw; 2 Department of Bioinformatics and Medical Engineering, Asia University, Taichung City 41354, Taiwan; sethho@asia.edu.tw; 3 School of Medicine, China Medical University, Taichung City 40447, Taiwan; D33977@mail.cmuh.org.tw (J.-J.L.); hooiyeen@gmail.com (H.Y.N.); 4 Department of Plastic and Reconstruction Surgery, China Medical University Hospital, Taichung City 40447, Taiwan; 5 3D Printing Medical Research Institute, Asia University, Taichung City 41354, Taiwan; 6 x-Dimension Center for Medical Research and Translation, China Medical University Hospital, Taichung City 40447, Taiwan; momoyin911@gmail.com; 7 Graduate Institute of Biomedical Sciences, China Medical University, Taichung City 40447, Taiwan

**Keywords:** gelatin-methacryloyl, fibroblast, extracellular matrix remodeling, 3D-bioprinting

## Abstract

Gelatin-methacryloyl (GelMa) is a very versatile biomaterial widely used in various biomedical applications. The addition of methacryloyl makes it possible to have hydrogels with varying mechanical properties due to its photocuring characteristics. In addition, gelatin is obtained and derived from natural material; thus, it retains various cell-friendly motifs, such as arginine-glycine-aspartic acid, which then provides implanted cells with a friendly environment for proliferation and differentiation. In this study, we fabricated human dermal fibroblast cell (hDF)-laden photocurable GelMa hydrogels with varying physical properties (5%, 10%, and 15%) and assessed them for cellular responses and behavior, including cell spreading, proliferation, and the degree of extracellular matrix remodeling. Under similar photocuring conditions, lower concentrations of GelMa hydrogels had lower mechanical properties than higher concentrations. Furthermore, other properties, such as swelling and degradation, were compared in this study. In addition, our findings revealed that there were increased remodeling and proliferation markers in the 5% GelMa group, which had lower mechanical properties. However, it was important to note that cellular viabilities were not affected by the stiffness of the hydrogels. With this result in mind, we attempted to fabricate 5–15% GelMa scaffolds (20 × 20 × 3 mm^3^) to assess their feasibility for use in skin regeneration applications. The results showed that both 10% and 15% GelMa scaffolds could be fabricated easily at room temperature by adjusting several parameters, such as printing speed and extrusion pressure. However, since the sol-gel temperature of 5% GelMa was noted to be lower than its counterparts, 5% GelMa scaffolds had to be printed at low temperatures. In conclusion, GelMa once again was shown to be an ideal biomaterial for various tissue engineering applications due to its versatile mechanical and biological properties. This study showed the feasibility of GelMa in skin tissue engineering and its potential as an alternative for skin transplants.

## 1. Introduction

Burn injuries are one of the most common types of cutaneous wounds in the world. Healing often involves the regeneration of the epidermis and its involved connective tissues [1]. In addition, wounds typically first undergo re-epithelization before dermis regeneration. Therefore, for patients with burn injuries, sufficient re-epithelization not only enhances tissue regeneration, but it also acts as a barrier to prevent soft tissue infections and moisture loss. The current gold standard for the repair of burn injuries is also in good agreement with the above-mentioned rule. Covering a burn wound with autologous skin grafts harvested from a non-injured site is the currently accepted standard for burn injuries. However, more often than not, there is insufficient healthy skin for harvesting, and autografts mean that the patient has to undergo multiple surgeries for the purpose of harvesting and grafting, thus exposing the patient to more risks. Therefore, various types of tissue-engineered scaffolds have been developed since the past decade, and there are currently several skin substitute products on the market [2].

An ideal skin substitute should have biological functions, such as supporting cell proliferation and differentiation, and should also have sufficient mechanical properties to withstand transplant and surgical conditions [3]. Currently, skin substitutes on the market can be broadly categorized according to their material and cellularity [4]. Dermagraft^TM^ is a widely used skin substitute in clinical applications that is made up of mainly fibroblasts seeded in a specific matrix. Apligraf™ and OrCel™ are bilayer collagen sponges with fibroblasts and keratinocytes. Fibroblasts are commonly seeded into the scaffold because they have the ability to secrete and degrade proteins during skin reconstruction and regeneration, thus leading to extracellular matrix (ECM) remodeling. Furthermore, fibroblasts can secrete various growth factors and cytokines, which in turn lead to enhanced regeneration and healing [5]. However, it is worth noting that even with our current knowledge and technology, a full-thickness skin substitute with appropriate vascularization is still currently not available. Furthermore, despite good clinical results, several critical problems still remain to be solved. A majority of the available skin substitutes contain allogenic skin that can induce immune responses [6]. In addition, there is no perfect sterilization method that can irradiate and rid the substitutes of all pathogens.

The emergence of 3D printing technology has made it possible to fabricate 3D structures with control over the internal architecture and geographical properties [7]. The fate of a cell is greatly influenced by the environment that it resides in, which consists of an ECM and its attached protein motifs [8]. Many natural and synthetic materials used for tissue engineering have emerged in the past decade in efforts to create an ideal microenvironment for cells [9], among which gelatin has gained a certain amount of attention because it is derived from collagen, and collagen is an important component of the ECM [10]. Cells embedded in gelatin scaffolds were shown to have enhanced biological characteristics as compared to synthetic materials [11]. In addition, gelatin has been shown to have good biodegradability, biocompatibility, and has high cell/tissue affinity, which allows efficient tissue integration between scaffolds and native bone [12]. Hydrogels made from gelatin have a 3D macromolecular network that has a high-water content [13,14]. This characteristic not only enhances biocompatibility, it adds elasticity to hydrogels and thus makes it possible to better mimic native ECM [15,16,17]. However, gelatin-based hydrogels have poor mechanical integrity and have thermo-reversible properties at body temperature, which severely limit their potential for clinical application s [18,19].

Over the last few decades, light-curable polymers have become an attractive strategy employed in the construction of hydrogels for tissue engineering applications [20,21]. Methacrylic anhydride (Ma) has been used to modify gelatin by substituting methacryloyl substitution groups onto the reactive amine so as to obtain photoreactive gelatin methacrylate (GelMa), which can be crosslinked when exposed to UV light [22,23,24]. In addition, the formulations of light-curable polymers are easy to develop. Polymers work as precursors that remain stable and can be formed into defect shapes to fabricate patient-specific implants, which in turn enhances the mechanical properties and integration of both scaffolds and tissues [25]. The structural integrity and mechanical properties of GelMa hydrogels depend on the degree of crosslinking, composition, or matrix concentrations [26]. As technology advances, there is a need to explore and look into the interactions between scaffolds and cells so optimized scaffolds for different tissues can be fabricated [27,28,29,30]. Other factors, such as photo-initiator concentrations, UV cross-linking strength, and duration were recently investigated to determine their effects on human primary cells and thus would remain as a constant in this study [19,31].

In this study, 5%, 10%, and 15% GelMa hydrogels were fabricated, and physical properties such as swelling, degradation rate, and sol-gel transition temperature were assessed. Furthermore, mechanical properties such as stiffness and its effects on cellular behavior were also evaluated. Human fibroblasts (hDF) were used in this study and were encapsulated in different concentrations of GelMa with varying mechanical properties. This study was aimed at investigating the effects of gelatin concentrations on cellular behavior in order to obtain optimal parameters for the fabrication of skin tissues. Simultaneously, we optimized the printing parameters for the different GelMa hydrogels and fabricated GelMa scaffolds that could be applied in future skin tissue engineering studies.

## 2. Materials and Methods

### 2.1. Synthesis of Gelatin-Methacryloyl

To synthesize gelatin-methacryloyl, we used type B gelatin (porcine, Sigma-Aldrich, St. Louis, MO, USA) and dissolved it in phosphate-buffered saline to obtain 10% *w/v* gelatin solutions. An amount of 0.6 g of methacrylic anhydride (Ma, Sigma-Aldrich, St. Louis, MO, USA) per gram of gelatin was dripped into the solution and continuously stirred at 400 rpm at 50 °C for 3 h to allow modification. Then, warm phosphate-buffered solution was added into the gelatin-methacryloyl solution, which was then centrifuged to remove any excess unreacted substances and dialyzed for 7 days to obtain dry gelatin-methacryloyl (GelMa) foam. All foams were stored at −20 °C.

### 2.2. Determination of GelMa Degree of Substitution

To determine the degree of substitution, the alternation in the amounts of primary amino functional groups of gelatins before and after methacrylation was quantitatively measured as suggested by a previous report [32]. To this end, a ninhydrin test (Sigma-Aldrich, St. Louis, MO, USA) was used according to the manufacturer’s instructions. Both gelatin and GelMa were dissolved in deionized water, after which ninhydrin solution was dripped into the solutions and allowed to react for 10 min in an oven. An amount of 500 μL of pure ethanol was used to stop the reaction, and the absorbance was measured at a wavelength of 570 nm. Glycine, with its known wavelength among the various amino groups, was used as the comparison group. In addition, 1H NMR was used to determine for the degree of substitution. Both gelatin and GelMa were dissolved in deuterium oxide (Sigma-Aldrich) and analyzed using a 400 MHz NMR spectrometer (JEOL Co. Ltd., Tokyo, Japan). The degree of substitution was calculated using the following formula [33]:

(1) $$\mathrm{Methacrylation}\;\mathrm{degree}\;\left(\%\right)=\frac{\mathrm{Number}\;\mathrm{of}\;\mathrm{methacrylate}\;\mathrm{groups}}{\mathrm{Number}\;\mathrm{of}\;\mathrm{amine}\;\mathrm{group}\;\mathrm{on}\;\mathrm{unreacted}\;\mathrm{polymers}}\times \;100$$

### 2.3. Preparation of GelMa Hydrogels

The GelMa hydrogels were prepared using an extrusion-based Inkredible+ bioprinter 3D printer (Cellink, Gothenburg, Sweden). The foams were first dissolved in phosphate-buffered solutions and 0.5% *w/w* of Irgacure 2959 photo-initiator (Sigma-Aldrich, St. Louis, MO, USA) was added to allow photo-curing. Pluronic^®^ F-127 (F127, Sigma-Aldrich, St. Louis, MO, USA) was first used as the supporting frame for the GelMa hydrogels. In this study, F-127 was first extruded via the bioprinter before the hydrogels were deposited into the molds. Then, the F-127 and GelMa hydrogels were exposed to 10 mW/cm^2^ 365 nm UV (Spot Cure Series, SP11, Ushio, Japan) at a distance of 30 cm for 60–120 s. To obtain the GelMa hydrogels, the F-127 GelMa hydrogel was immersed in cold water to allow dissolution of the F-127 support.

### 2.4. Hydrogel Swelling Analysis

To determine the extent of the swelling behavior in the hydrogel, different concentrations of GelMa hydrogels were first deposited into F127-printed molds with a pre-determined outer diameter of 12 mm and a thickness of 1 mm, after which the hydrogels were cured under UV lighting and left to incubate in phosphate buffer solution (PBS, Invitrogen, Carlsbad, CA, USA) for different time intervals. At the different time points, the hydrogels were removed from the PBS solution, dried using filter paper, and weighed to obtain the wet weight (Ws) of the specimens. Subsequently, the hydrogels were lyophilized and weighed again to obtain the dry weight (Wd) of each specimen, after which the swelling ratio was calculated using the following formula:

(2) $$\mathrm{Swelling}\;\mathrm{ratio}\left(\%\right)=\frac{\left(\mathrm{Ws}–\mathrm{Wd}\right)}{\mathrm{Wd}}\times 100\%.$$

### 2.5. Rheological Measurement

The rheological properties of the GelMa prepolymer solution at different concentrations were characterized using a rheometer (MCR 302, Anton Paar, Graz, Austria) equipped with a cone-plate geometry probe (CP50-1; diameter: 50 mm, cone angle: 1°, cone−plate gap: 0.101 mm). An amount of 0.7 mL of the prepolymer samples was loaded on the plate at 40 °C to fully fill the gaps between the geometry holders. The storage modulus (G’) and loss modulus (G’’) were recorded at an oscillating frequency of 1 Hz and a strain of 0.25%, with temperature ramping from 40 to 10 °C at a cooling rate of 1 °C /min. The gelling temperature of the prepolymer solutions was defined as the temperature at which the value of G’ was higher than that of G’’.

### 2.6. Mechanical Testing

The mechanical properties of the GelMa hydrogels were measured using tensile tests conducted with the EZ-Test machine (Shimadzu, Kyoto, Japan). The specimens were printed into the shape of a dumbbell with a thickness of 2 mm. Similarly, F127 was used as the support structure for the GelMa. For the test, a tensile pull at a constant rate of 1 mm/min was applied to the hydrogel until the hydrogel tore completely, and the corresponding stress and strain were then recorded upon the complete lesion of the hydrogels. The Young’s modulus was used to calculate the crosslink densities, which represents the mole number of active chain segments per unit volume (m^3^) of each hydrogel group by using the rubber elasticity theory [34] according to the following formula:

(3) $$\mathrm{Crosslink}\;\mathrm{Density}=\frac{E}{3\mathrm{RT}}$$

Whereas *E*, R, and T stand for the Young’s modulus in Pascal (Pa), universal gas constant in (8.3144 J/mol·K), and absolute temperature (K), respectively.

### 2.7. Enzymatic Degradation of Hydrogels

GelMa hydrogels with an 8 mm diameter and 1 mm thickness were fabricated using the methods described above. The fabricated hydrogels were placed and left in phosphate-buffered solution for 24 h to obtain swelling equilibrium. Then, collagenase was placed into the solution and left to incubate for 2, 4, 12, 24, 72 h and 1 and 2 weeks. At the various time points, the swollen hydrogels were washed, lyophilized, and weighed. Six samples were used at each time point to obtain the averages and standard deviations. Lastly, degradation rates were calculated using the following formula:

(4) $$\mathrm{Degradation}\;\mathrm{Rate}\;\left(\%\right)=\frac{W0-\mathrm{Wt}}{W0}\times \;100\%.$$

### 2.8. Culture of Fibroblasts in Hydrogel

Human dermal fibroblasts (hDF; ScienCell Research Laboratories, Carlsbad, CA, USA) were used in this study for the subsequent in vitro studies. The hDF were cultured in Fibroblast Medium (ScienCell Research Laboratories) with the respective recommended antibiotics. The cells were trypsinized using trypsin/EDTA, collected using a hemocytometer, and placed into GelMa solution at a density of 2 × 10^6^ cells/mL. Then, the GelMa hydrogels were fabricated using the methods described above and cultured in fibroblast medium.

### 2.9. Live/Dead Staining and Cell Viability

After 0, 1, 3, and 7 days of culture, the cell-laden hydrogel disks were washed in PBS and then incubated with calcein-AM (2 µM) and propidium iodide (4 µM) for 30 min. After that, the stained live/dead cells in the hydrogels were observed under a confocal microscope (Leica TCS SP2, Leica, Heidelberg, Germany). To investigate cell viability, the cell-laden hydrogels were first digested using 3 mg/mL collagenase (Sigma-Aldrich) at 0, 1, 3, and 7 days of culture, after which the cells were collected, and 18 μL of each cell sample was stained with 2 μL of Acridine Orange/Propidium Iodide (AO/PI) (Logos Biosystems, Annandale, VA, USA). An amount of 10 μL of the mixed cell sample was then loaded into the inlet of the counting slide chamber and left for 10 sec to allow the cells to settle down. The number of live/dead cells was counted using a LUNA-FLTM Automated Fluorescence Cell Counter (Logos Biosystems). Live cells were seen as green fluorescence, and apoptotic/necrotic cells were stained with red fluorescence.

$$\mathrm{The}\;\mathrm{cell}\;\mathrm{viability}\;\mathrm{was}\;\mathrm{defined}\;\mathrm{as}:\;\frac{\mathrm{Number}\;\mathrm{of}\;\mathrm{live}\;\mathrm{cells}}{\mathrm{Number}\;\mathrm{of}\;\mathrm{total}\;\mathrm{cells}\;}\times 100\%.$$

### 2.10. Cytoskeleton Staining

F-actin stains were used to observe for the cytoskeleton of hDF after 1, 3, and 7 days of culture. The cell-laden hydrogels were washed three times with PBS for 10 min, fixed with 4% paraformaldehyde at room temperature for 30 min, and further washed three times with PBS. Next, 0.25% of Triton X-100 was used to permeabilize the cells, which were then rinsed with PBS and blocked with 1% bovine serum albumin (BSA, Gibco, Gaithersburg, MD, USA). In this study, the actin filaments were stained using Alexa Fluor 594 phalloidin (Invitrogen, Carlsbad, CA, USA), and the cell nuclei were stained with 300 nM 4’,6-diamidino-2-phenylindole (DAPI, Invitrogen). PBS was used to wash the stained specimens, which were then viewed using a confocal microscope (Leica TCS SP2, Leica, Wetzlar, Germany).

### 2.11. Western Blot Analysis

The hDF were loaded into hydrogels and collected based on similar methods to those described above. The collected cells were then washed with cold phosphate-buffered solution and treated with radioimmunoprecipitation assay buffer (Abcam, Cambridge, UK) and cold centrifuged a 14,000 rpm for 20 min. A Bradford protein assay from Bio-Rad, Richmond, CA, USA was used to evaluate for the levels of the different proteins. According to the manufacturer’s instructions, SDS-PAGE was used to separate the proteins, which were subsequently transferred onto polyvinylidene difluoride membranes. Target primary antibodies (β-actin, Abcam; MMP2, Millipore, Billerica, MA, USA; MMP9, Abcam; Decorin, Abcam) were placed onto the membranes and incubated overnight. Then, the membranes were washed and incubated with either horseradish peroxidase-conjugated anti-rabbit IgG (1:2,000 dilution; Genetex, Hsinchu, Taiwan) or horseradish peroxidase-conjugated anti-mouse IgG (1:2,000 dilution; Genetex) for 1 h at room temperature. The Fusion-Solo chemiluminescence system (Vilber, Paris, France) and ECL Western blotting Detection Reagents (Thermo Fisher Scientific, Waltham, MA, USA) were then used to detect the signals emitted from the samples.

### 2.12. Immunocytochemistry

After 14 days of culture, the cell-laden hydrogels were washed with PBS and fixed with 10% neutral formalin for 2 days. After that, a tissue processor (Leica ASP300 S, Heidelberg, Germany) was used to dehydrate the samples, followed by paraffin fixation. Then, the embedded samples were sectioned to obtain cross-sections 4 µm thick. The cross-sections were deparaffinized, stained with Haemotoxylin and Eosin (HE) stainning, and examined using a microscope. In addition, the immunohistochemical expression of MMP2, MMP9, and collagen I were analyzed for cellular proliferation quantification. Briefly, the tissue section was deparaffinized and rehydrated with xylene and a series of ethanol solutions. The tissue sections were treated for 15 min with a hydrogen peroxide block and protein block solutions from the UltraVision Quanto Detection System (Thermo Fisher Scientific). The slides were then washed three times with TBST and incubated with a primary antibody amplifier and HRP polymer (UltraVision Quanto, Thermo Fisher Scientific) for 10 min. Finally, the sections were stained using 3’-Diaminobenzidine (DAB) and then counter-stained with hematoxylin, dehydrated, and examined under a microscope. All images from the microscope were taken with a built-in camera (BX-53; Olympus, Tokyo, Japan).

### 2.13. Scaffold Fabrication

5%, 10%, and 15% GelMa solutions (code: G5, G10, and G15) containing photo-initiator 0.5% I2959 were blended homogeneously at 40 °C. Prior to printing, hDF (5 × 10^6^ cells/mL) were mixed with the GelMa solution by gently pipetting to avoid bubble formation. For complete mixing, the hDF-laden bioink was transferred into a 3-mL syringe with a needle gauge of 27 G before cooling. The GelMa scaffolds (20 × 20 × 3 mm^3^) were printed using an extrusion-based 3D printer (BioX, Cellink, Gothenburg, Sweden). The printing environment was set at 22 °C with the printing bed set at 16 °C during bioprinting. A multilayer grid construct with a 150 μm layer distance was printed with an extrusion pressure of 100 kPa and a constant printing speed of 20 mm/s. Finally, the bioprinted constructs were further UV-crosslinked at a distance of 30 cm for 90 s.

### 2.14. Statistical Analyses

A minimum of three independent tests were performed for each experiment, and all data in this study were reported as the mean ± standard deviation (SD). A one-way ANOVA analysis of the between-group results was conducted. Statistical differences were set at *p* < 0.05 (*), and (**) and (***) indicated *p* < 0.01 and *p* < 0.001, respectively.

## 3. Results and Discussion

### 3.1. Determination of Degree of Methacrylation

**Figure 1:**
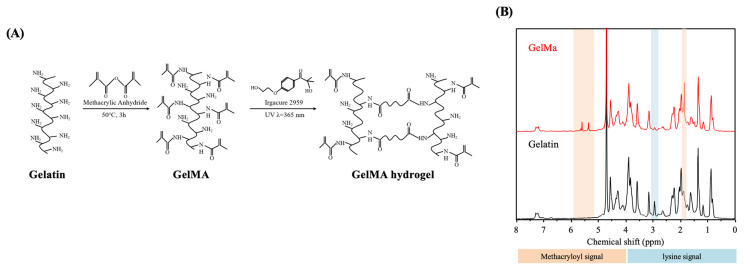
Synthesis of porcine Gelatin-methacryloyl (GelMa) and fabrication of a photo-crosslinked GelMa hydrogel. (**A**) Gelatin was reacted with methacrylic anhydride (Ma) to introduce a methacryloyl substitution group on the reactive amine and hydroxyl groups of the amino acid residues. GelMa photo-crosslinking to form a hydrogel matrix under UV irradiation. (**B**) ^1^H NMR spectra of unmodified gelatin and GelMa.

Gelatin has a polypeptide backbone with multiple available functional groups, such as amino, hydroxyl, and carboxyl groups, which can serve as grafting sites for additional molecules (Figure 1A). GelMa is synthesized via modification with a methacryloyl group (Ma), and the degree of methacrylation can be adjusted using different ratios of gelatin to MA to produce scaffolds with different mechanical properties. Furthermore, such modification makes it possible to retain the unique properties of gelatin and yet allows the viscous gelatin to be solidified via permanent crosslinking of the MA groups. The synthesized GelMa was found to have a degree of methacrylation of 82–85% in between batches (results not shown). Reproducibility is a crucial factor for GelMa because different degrees of methacrylation can affect properties such as swelling behavior, mechanical properties, and degradation rate. Therefore, the results indicated that the methodologies used were consistent and reliable [35]. Different sources of gelatin can be used for producing of GelMa. However, fish gelatin and type A and B porcine have slightly different blood factors and amino acid motifs, thus resulting in different degrees of substitution [31,36]. Among the various types of gelatin, porcine gelatin is the most used for tissue engineering due to its higher mechanical properties. The 1H NMR spectra were used to determine the successful modification of Ma into gelatin molecules. The results were shown in Figure 1B. It can be clearly observed that both gelatin and GelMa reveal complex spectra due to the presence of a wide range of amino acids in the polypeptide. It is of note that the successful methacrylation of gelatin can be achieved by the emergence of methyl and vinyl proton signals at δ = 1.9 and 5.4/5.7 ppm, respectively, along with the reduced intensity of the lysine signal at δ = 2.9 ppm. Additionally, the residue signals of GelMa shared similar chemical shifts and peak intensities to those of gelatin, indicating that the structure and integrity of the gelatin were still present, and thus, the ideal properties of gelatin could be still retained after modification. Chen et al. fabricated type A porcine GelMa with 1 M, 5 M, and 10 M of Ma and found that different amounts of Ma led to varied functionalization (49.8%, 63.8%, and 73.2%, respectively), thus leading to different mechanical and biological properties [37]. This result showed that the percentages of gelatin or MA could be fine-tuned to achieve different effects for a specific need or tissue type. In this study, 5%, 10%, and 15% gelatin (G5, G10 and G15) was used to fabricate GelMa to determine if this gelatin is feasible for skin tissue engineering.

### 3.2. Sol-gel Transition Temperature of Hydrogel

**Figure 2:**
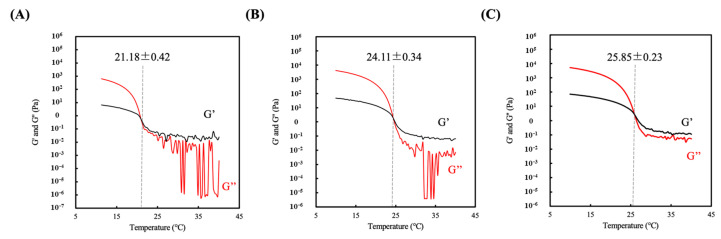
Rheological properties of the three hydrogel composites: (**A**) G5, (**B**) G10, and (**C**) G15. The cross point between storage moduli (G′) and loss moduli (G″) is regarded as the sol-gel transition temperature.

The measurement of the sol-gel transition temperature of the GelMa hydrogel solution was carried out via a rheometer equipped with a cone-plate geometry probe. The samples were cooled from 40 °C to 10 °C, and the storage modulus (G’) and loss modulus (G’’) were recorded as measurements of the sol-gel transition characteristics. A constant frequency (1 Hz) and strain (0.25%) were applied to the different GelMa concentrations (5–15 *w/v* %). As expected, an increase in GelMa concentration led to higher G’, as shown in Figure 2. The point where G’ intersected with G’’ was determined as the sol-gel transition temperature of G5, G10, and G15, which was found to be 21.18 ± 0.42, 24.11 ± 0.34, and 25.85 ± 0.23 °C, respectively. This sol-gel transition temperature is a critical component for bioprinting of hydrogels scaffolds because the printing temperature affects scaffold quality. Temperatures higher than the sol-gel temperature will cause GelMa to liquify, and temperature lower than the sol-gel temperature will cause over-gelation, thus creating a problem with printability [38].

### 3.3. Swelling Characteristics

**Figure 3:**
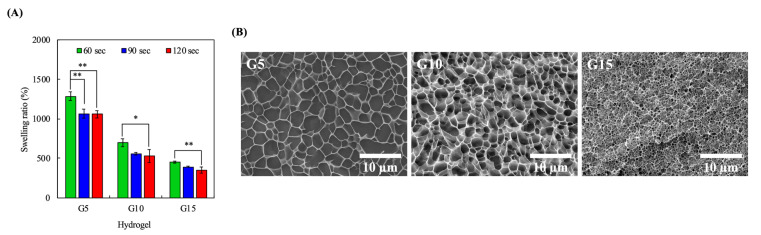
(**A**) Equilibrium swelling properties of the GelMa hydrogels. Error bars represent the SDs of measurements of six samples. (*) and (**) indicated *p* < 0.05 and *p* < 0.01, respectively. (**B**) Cryo-imaging of the hydrogel microstructure. The G5, G10, and G15 samples were exposed to 365 nm UV light at a distance 30 cm for 90 s.

The swelling capability of a hydrogel is an important factor that indirectly indicates its water absorption capacity and thus, it can be used to predict the rate of degradation. The degree of cross-linking and pore-sizes are some of the factors that can influence swelling capability. It is important to note that the swelling capacity and degradation rate of scaffolds are important aspects of a good hydrogel because these factors can affect healing. As shown in Figure 3A, it was found that increasing GelMa concentrations led to significantly reduced swelling at all durations of UV curing. Furthermore, it can be seen that the 5% hydrogels exhibited a significant decrease in swelling ratio at all time points. However, the 10% and 15% hydrogels only had significant differences between 60 s and 120 s. None of the three groups showed significant differences between 90 s and 120 s, so 90 s was set as the curing duration for the subsequent studies. Furthermore, it could be indirectly inferred that the 15% GelMa had a slower degradation rate as compared to the rest of the groups with 90 s of UV curing. This was hypothesized to be due to the higher cross-linking densities of G15. To prove this point, cryo-SEM images of the hydrogels were taken after exposure to 365 nm UV for 90 s at a distance of 30 cm in order to look for any internal structural differences among the hydrogels. As shown in Figure 3B, higher concentrations of GelMa had denser internal architectures with smaller pores, thus indicating that there was higher cross-linking in groups with higher GelMa concentrations. However, there was a second-order response between the pore diameter and the swelling ratio of the hydrogel, which could possibly be attributed to the fact that GelMa at a higher concentration causes the formation of inner polymer networks characterized by greater stiffness, thus resulting in the remarkable restriction of the water molecules [39].

### 3.4. Mechanical Properties

**Figure 4:**
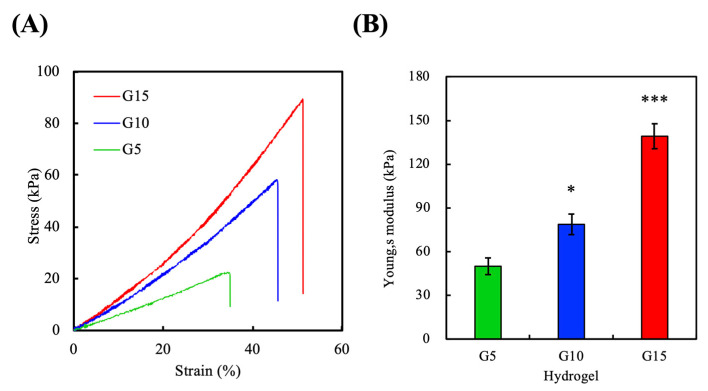
Mechanical characterization of the GelMa hydrogels. (**A**) Stress–strain curves of the G5, G10, and G15 samples. (**B**) Young’s moduli (average ± standard deviation) for G5, G10, and G15. (*) and (***) indicated *p* < 0.05 and *p* < 0.001, respectively.

The mechanical properties of scaffolds have been shown to affect cell function, ECM remodeling, and cellular differentiation [3]. To determine the mechanical properties of the various GelMa hydrogels, an elastic modulus test was performed on dumbbell-shaped specimens exposed to 90 s of curing time. The tensile stress-strain curves of the hydrogels are shown in Figur 4A, and the results of the calculated Young’s modulus are shown in Figure 4B. The stress-strain curves demonstrated a positive correlation between the GelMa concentrations and the mechanical properties of the scaffolds, ranging from 22.1 kPa for G5 to 88.9 kPa for G15. Similarly, it was hypothesized that the increase in stress-strain was due to the increased cross-linking densities at higher GelMa concentrations. Better mechanical properties have been shown by others to be favorable for keratinocyte growth. Furthermore, increased elastic moduli is thought to be beneficial for surgical manipulations and for implantations. The young’s moduli of G5, G10, and G15 were 49.9 ± 5.7 kPa, 78.7 ± 7.0 kPa, and 139.1 ± 8.6 kPa at room temperature, respectively, which were in sync with our tensile stress–strain results given above. In addition, G15 exhibited a steeper slope with a higher strain at break than other groups, indicating that it could withstand sudden stretching or bending that would prevent the occurrence of premature failures after implantation. Moreover, the calculated crosslink density of G5, G10, and G15 were 6.7 ± 0.8, 10.6 ± 0.9, and 18.7 ± 1.2 mol/m^3^, respectively.

### 3.5. Degradation

**Figure 5:**
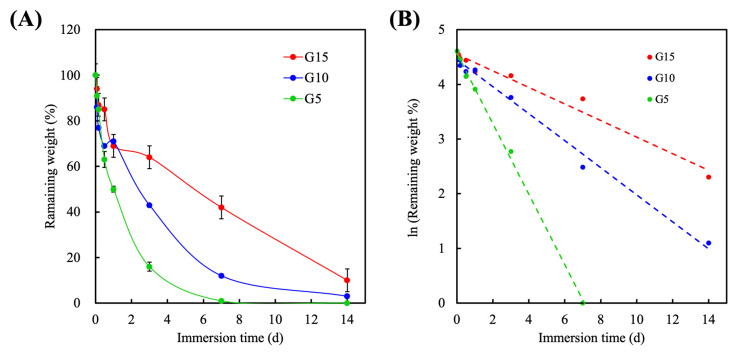
(**A**) Enzymatic degradation of the GelMa hydrogels in the collagenase-contained PBS at 37 °C and (**B**) the first-order kinetic plot for the degradation rate of the hydrogels.

To evaluate degradability, the GelMa hydrogels were incubated in collagenase solutions, and the degradation rates of the various hydrogels are shown in Figure 5A. The hydrogels were soaked in 2 units∙mL^-1^ of collagenase solution for 2, 4, 12, 24, 72 h, and 1 and 2 weeks. As shown, the degradation rate decreased with increases in the GelMa concentration, with G5 showing complete degradation by 7 days. At 24 h, G5 had a remaining weight percentage of 50% ± 1.3%; G10 was at 71% ± 0.7%, and G15 was at 69% ± 1.6%. After 72 h of immersion, the remaining weight percentages of G5, G10, and G15 were 16% ± 2.0%, 43% ± 0.1%, and 64% ± 1.6%, respectively. All hydrogels had a constant degradation rate, with the exception of G15, which exhibited steeper degradation for the first 2 days as compared to the other samples. After 14 days of immersion, G10 and G15 had approximately 4% and 8% of remaining weight, respectively. It was hypothesized that the constant degradation rate was due to the presence of MMP-sensitive motifs in the GelMa [40]. Furthermore, the increased Ma cross-links in the G15 hydrogels were hypothesized to be responsible for the slower degradation because the crosslinks were resistant to collagenase. The rate of degradation is an important factor because there is a need to ensure optimal healing before complete degradation. In our study, we conducted the in vitro degradation test by incubating the photocured hydrogels with an excess amount of collagenase. The correlation curves of the natural log of remaining weight percent against incubation time in all groups were approximately linear with R^2^ value of 0.9979, 0.9896, and 0.9715 for G5, G10, and G15, respectively, indicating that the degradation of the hydrogels followed the pseudo-first-order degradation kinetics (Figure 5B). The calculated degradation rate constant of G5, G10, and G15 were 0.645, 0.247, and 0.152 d^−1^, respectively. Furthermore, ensuring optimal healing before degradation can help avoid secondary infections. Despite the fact that the mechanical strength of the hydrogels can gradually reduce within time due to the enzymatic digestion, an appropriate degree of degradation in the GelMa allows encapsulated cells in the degrading GelMa hydrogels to secrete ECM and associated proteins, thus remodeling the micro-environment. Interestingly, in a previous study conducted by Yoon et al., it was revealed that 10% porcine and fish GelMa had a remaining weight percentage of 56.8% ± 2.2% and 96.3% ± 2.1%, respectively, after 9 h of collagenase treatment [36]. Through the cross-sectional SEM images, it could be observed that the fish GelMa microstructure had a collapsed, disorganized internal architecture while the porcine GelMa had well organized porous microstructures. The above results implied that mechanical and degradation properties could be easily fine-tuned by adjusting the ratio of gelatin and Ma. Our results demonstrated that the tensile strengths and completely enzymatical degradation periods of the GelMa hydrogels can be tuned in ranges between ~18 kPa to ~90 kPa and 7 to 14 days, respectively, by adjusting the concentration of the GelMa prepolymer. Therefore, this further indicated that GelMa hydrogels have a broad spectrum of capabilities that can be adjusted to suit specific requirements.

### 3.6. In Vitro hDF Culture

**Figure 6:**
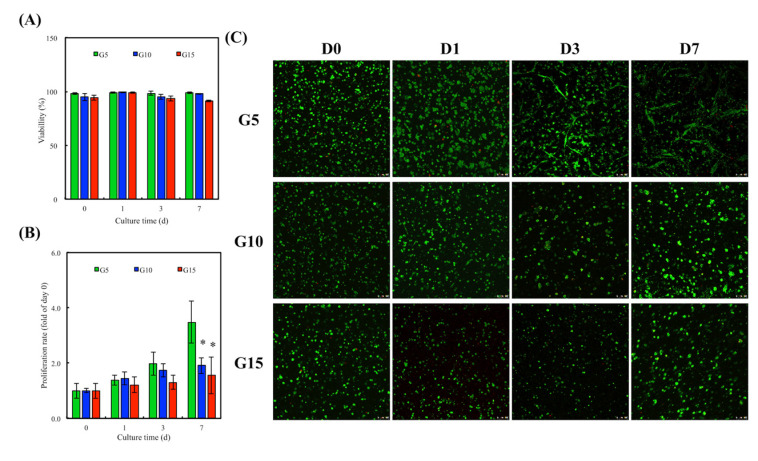
(**A**) Cell viability of human dermal fibroblast cells (hDF) encapsulated in GelMa hydrogel within 7 days of cell culture. (**B**) Proliferation rate and (**C**) live/dead staining of hDF cultured in the GelMa hydrogels for various time points. Scale bar = 100 µm. (Green: live cells; Red: dead cells). Error bars represent the SDs of the measurements of six samples. “*” indicates a significant difference (*p* < 0.05) compared to G5.

**Figure 7:**
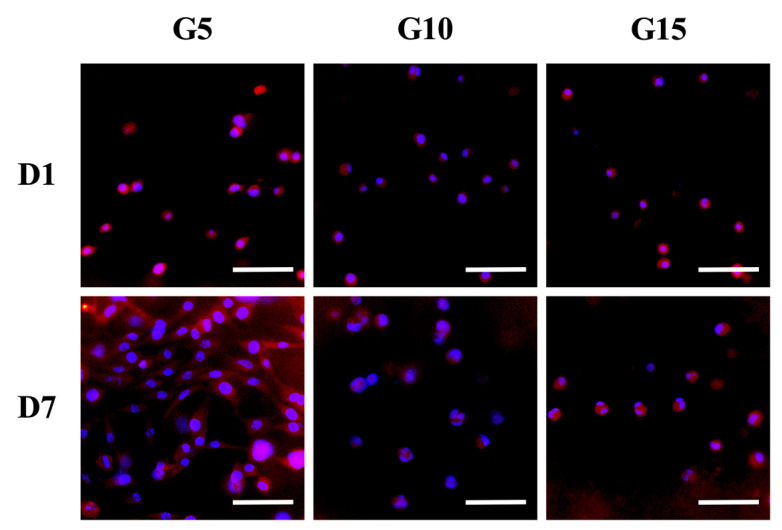
Cell spreading at the various concentrations of the GelMa constructs. Fluorescence images of hDF stained with F-actin (red) and cell nuclei (blue) after being cultured in GelMa hydrogels for 1 and 7 days, respectively. Scale bar: 200 µm.

hDF were encapsulated in GelMa hydrogels for a fixed period of time and collected using collagenase. The ability of cells to attach and spread on hydrogels is a basic requirement for subsequent tissue regeneration. Results have been published stating that the better the adherence and cell spreading, the better the proliferation and differentiation. The viability of the hDF was evaluated using quantification of live and dead cells on the different GelMa immediately after UV cross-linking. As shown in Figure 6A, G5 had a cellular viability of 98.2% ± 0.9%; G10 had a cellular viability of 94.7% ± 3.1%, and G15 had a cellular viability of 94.2% ± 2.4%. These initial results showed that the UV intensity used in this study did not evidently affect cell viability. In addition, the cell viability for subsequent days of in vitro culture was over 90% for all groups, thus further indicating the innate biocompatibility of GelMa hydrogels. The proliferation rate of the hDF in the hydrogels was assessed and is shown in Figure 6B. Interestingly, it should be noted that the hDF in the G5 groups had a significantly higher proliferation rate as compared to the G10 and G15 groups. It was hypothesized that G10 and G15 had smaller pore sizes due to their compact internal architecture, thus limiting cellular attachment and growth. This result was different from the results published by Zhang et al., in which they stated that increasing GelMa concentration could increase the proliferation of immortalized human keratinocytes [28]. However, a direct comparison between our studies could not be made because different cell lines and different concentrations of photo-initiators were used. Additionally, a higher level of enzyme susceptibility of G5 may be another reason for the better cell proliferation by which more spaces inside of the polymeric networks could be generated due to the occurrence of the enzymatic digestion by the encapsulated cells and thus facilitated the migration, proliferation, and remodelability of hDF. Figure 6C shows the live and dead assays for the various hydrogels, where it can be seen that there were only a few dead cells (red) in each group. Furthermore, it can be seen that the cells on G5 took up a larger area of the hydrogel as compared to the rest, thus further supporting the quantitative cellular proliferation results. Similarly, it can be seen from Figure 7 that the hDF in the G5 hydrogels were spread better as compared to the other two groups. On the other hand, cells in both the G10 and G15 groups were aggregated together, forming small clusters, thus implying that the cells were not adhering well to the hydrogel or not able to digest the surrounding hydrogels to create more space for cell spreading, migration, and proliferation [41]. Reports have been made stating that the quality of cell adherence to hydrogels is directly related to downstream cellular behavior [42]. Combining the results referenced above, we therefore concluded that a hydrogel with a higher GelMa concentration will have higher network density and stiffness and thus will down-regulate cellular spreading and proliferation.

### 3.7. Cell Remodeling

**Figure 8:**
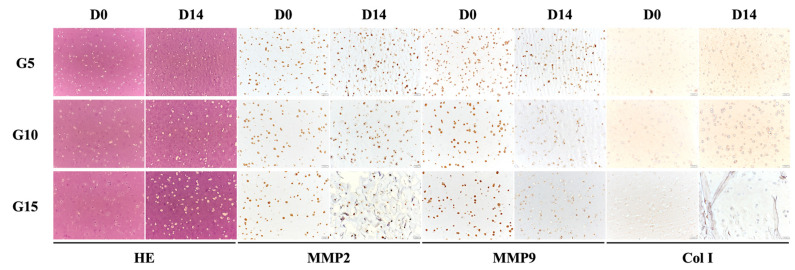
Histology characterization of embedded cell behavior in the GelMa hydrogel. hDF embedded in GelMa hydrogels at various concentrations were stained with HE, MMP2, MMP9, and collagen I 14 days after encapsulation.

From the immunohistochemical results shown in Figure 8, it can be seen that the cells on G5 were flattened after 14 days of culture as compared to G15. On the other hand, the HE staining showed that almost the entire visual field of the slide was covered with skin tissue, as indicated by the purple stain. In addition, it could be observed that the MMP-9 and Col I secretions on G5 were significantly higher than on the other samples after 14 days of culture, which concurred with the results discussed above. Type 1 collagen is one of the most abundant and important proteins in human beings. It assembles into fibers that then form the matrix for various kinds of tissues in the human body [43]. Together with the results above, it was concluded that the G5 hydrogel, in spite of the fact that it had weaker mechanical properties and degradation, was able to bring about enhanced cellular behavior that subsequently could lead to increased regeneration.

### 3.8. hDF-laden Scaffold Fabrication

**Figure 9:**
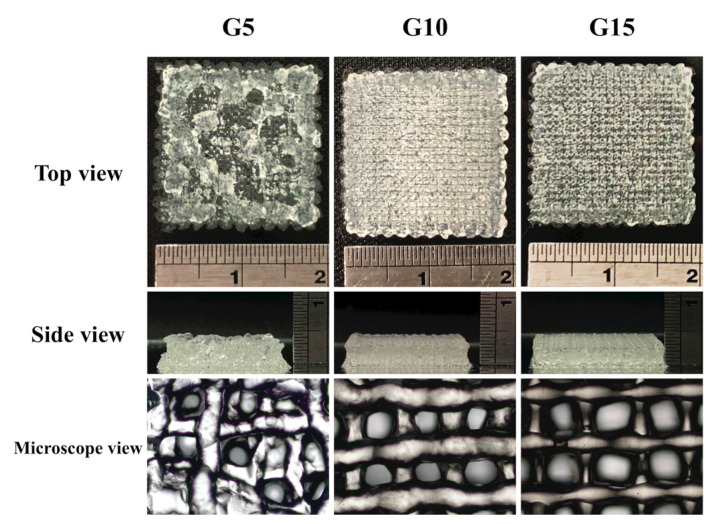
Bioprinting performance of GelMa bio-inks. Photographs showing the bioprinted scaffolds with top view, side view, and the microscope microstructure.

The skin is composed of two main layers: the epidermis, which is made up of tightly knitted epithelial cells and the dermis, which is made up of dense connective tissues that support the various associative structures, such as hair follicles and glands [44]. Based on the sol-gel transition temperature obtained from the rheology testing, we used extrusion-based 3D bioprinting to test the printability of G5, G10, and G15 bio-inks. There were three gelation statuses for the printed bio-ink: (i) under-gelation: The bio-ink tends to form a droplet at the nozzle tip, and will not form a complete structure even after extrusion; (ii) proper-gelation: The bio-ink can be extruded from the nozzle as an individual filament and stacked; (iii) over-gelation: The bio-ink is extruded as an irregular, discontinuous filament [45]. Images of the printed hydrogels are shown in Figure 9. The scaffolds had a dimension of 20 × 30 × 3 mm^3^, and it could be seen that G10 and G15 scaffolds could be printed easily using similar printing parameters. However, it was rather impossible to stack the G5 scaffolds due to the viscosity of the hydrogel. The printed filaments tended to fuse with one another once it was extruded out of the nozzle; thus, it could be seen from the side view that the G5 scaffolds were not as stable as the rest of the scaffolds. In this study, we synthesized various concentrations of GelMa hydrogels, and it was found that properties, including mechanical strength, degradation rate, and biological characteristics can be fine-tuned to suit each individual. However, specific modifications should be involved for reinforcing G5 without affecting its degradability because the weak mechanical strength and relative low gelation temperature of G5 can dramatically jeopardize printability, resulting in the poor structural control of the printed scaffolds. Chen et al. demonstrated that the mechanical strength of gelatin hydrogels can be reinforced 2.7-fold by introducing reinforcing fillers composed of deacetylated chitin nanofibers [46], which could be considered as an effective route to optimize the printability and enzymatic degradability of gelatin-based bioinks.

### 3.9. Cell Proliferation

**Figure 10:**
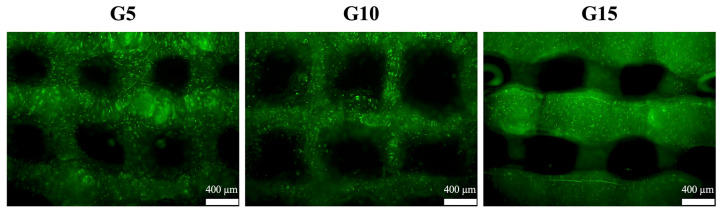
Image of the live/dead assay results for the hDF-laden GelMa scaffolds after fabrication. Scale bar: 400 µm.

**Figure 11:**
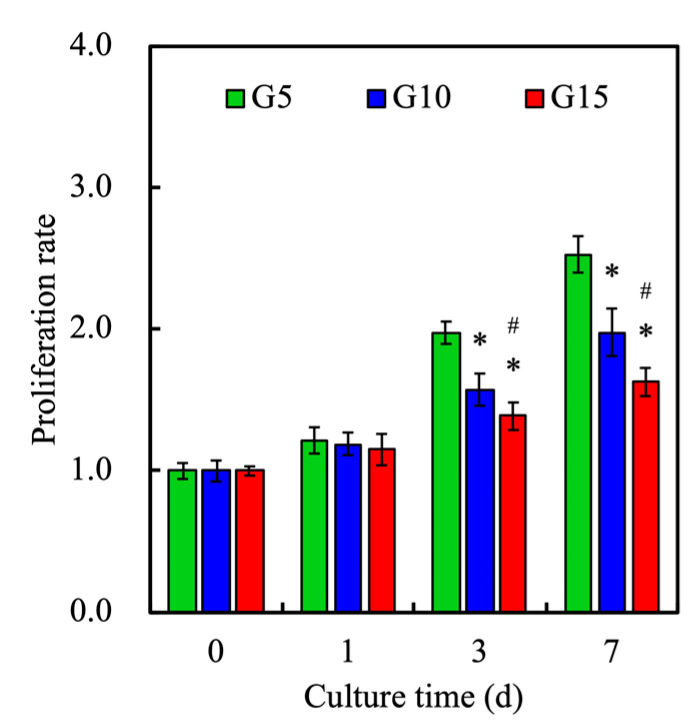
Cell proliferation rate of various hDF-laden GelMa scaffolds at various time-points. Data presented as mean ± SEM, n = 6 for each group. * indicates a significant difference (*p* < 0.05) from the G5 group. # indicates a significant difference (*p* < 0.05) from the G10 group.

After the fabrication of the hDF-laden GelMa scaffold, the cells were still alive (green, as shown in Figure 10). This also proves that the hDF-laden GelMa scaffolds will not be subjected to the hydrogel curing process and cause cell death after printing. In addition, the proliferation of hDF at different GelMa concentrations was quantified, for which the results are shown in Figure 11. It can be seen that the proliferation rate was inversely related to increasing concentrations of GelMa. This result was in good agreement with the results discussed above. Wu et al. showed that cells cultured in higher concentrations of GelMa had lower adhesion rates, and growth cones can sense substrate stiffness through the actin cytoskeleton and interact with the myosin polymerized at its terminal to form retrograde fibrillar actin [47]. Furthermore, our results indicated that the viability of hDF strongly depends on the substrate. The GelMa concentrations were significantly improved on G5 with a Young’s modulus of 49 KPa, as compared to the stiffer hydrogels. It was thus concluded that the G5 scaffold is the optimal hDF for skin regeneration. However, the main disadvantage of G5 lies in its weak mechanical properties.

### 3.10. Protein Expression of hDF-laden Scaffold

**Figure 12:**
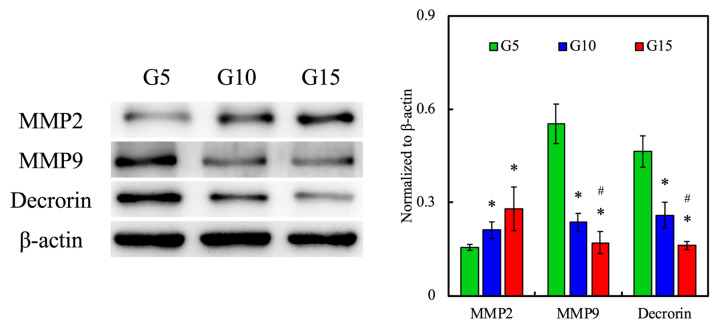
The in vitro effects of the extracellular matrix (ECM) remodeling marker (MMP2, MMP9, and decorin) of hDF-laden hydrogels with different concentrations of GelMa in the scaffolds. * indicates a significant difference (*p* < 0.05) from the G5 group. ^#^ indicates a significant difference (*p* < 0.05) from the G10 group.

To determine whether the GelMa hydrogels can support the cellular remodeling of hDF, we analyzed various protein expressions of cell-laden hydrogels using immunofluorescence staining, Western blotting, and immunocytochemistry. MMPs are zinc-containing endopeptidases with a broad range of substrate specificity. They are secreted by keratinocytes and dermal fibroblasts in response to multiple stress stimuli, such as UV radiation or inflammatory cytokines. There are five main subgroups of MMPs, of which MMP-2 and 9 are known as gelatinases. As their name suggests, they are involved in degrading a number of ECM components such as collagen type I and IV and are thus involved in skin regeneration and even angiogenesis [48]. The expression of MMP-2 and 9 was detected using immunofluorescence and Western blotting, for which the results are shown in Figure 12. Interestingly, the expression of MMP-9 was inversely related to increases in the GelMa concentrations, and there was an increase in the expression of MMP-2 with increasing concentrations. Clinically speaking, it was reported that elevated ratios of MMP-2/MMP-9 are related to the progression of invasive and metastatic tumors [49]. Therefore, even though both MMP-2 and 9 are known to be related in terms of regeneration, further studies are needed to determine the relationship of the various MMPs to skin regeneration. In addition, levels of decorin were also assessed. It could be seen that the expression of decorin decreased with increasing GelMa concentrations, which was similar to the case of MMP-9. Decorin is a component of the cellular matrix and is commonly found binding to type I collagen fibrils [50]. From the above observation, it was hypothesized that increasing concentrations of gelatin led to increased secretion of MMP-2, which then led to increased remodeling, seen in the form of lower levels of decorin in our results. However, there is a need to explore the relationships between the two MMPs to determine their combined effects in skin regeneration. Similarly, the quantification results in Figure 11 showed that G10 and G15 had significantly higher levels of MMP-2 as compared to G5 and significantly lower levels of MMP-9 and decorin as compared to G5.

## 4. Conclusions

In this study, we explored the gelatin methacryloyl concentrations of bio-inks for application in skin constructs. Hydrogel bio-inks used for successful bioprinting must exhibit desirable characteristics, such as good printability, minimal swelling and contraction, and excellent biocompatibility. In this study, the physical properties of GelMa were tested, where the relationship between concentration and stiffness was tested and assessed. The fabricated scaffolds showed different mechanical properties that depended on the ratio of the GelMa ink used. This test assessed variables and factors that could affect GelMa printability, and most importantly, it was found that cell-laden GelMa constructs showed ECM remodeling biological characteristics. The GelMa bio-ink systems also demonstrated tunability that has the potential to further enhance the quality of skin recovery. Most importantly, this study showed that it is possible to print GelMa hydrogels without the need for viscosity-enhancing additives. Therefore, we believe the current approach to a bioprinted-skin scaffold can be potentially applied to other cell-laden matrices and used in potential applications for skin regeneration.
